# Supplementary figures and images for: Phenological cycles in the Pantanal woody communities: Responses to climate and soil moisture seasonality
Source: PLoS One. 2025 Feb 7;20(2):e0316011. doi: 10.1371/journal.pone.0316011 (PMC11805406; doi:10.1371/journal.pone.0316011)

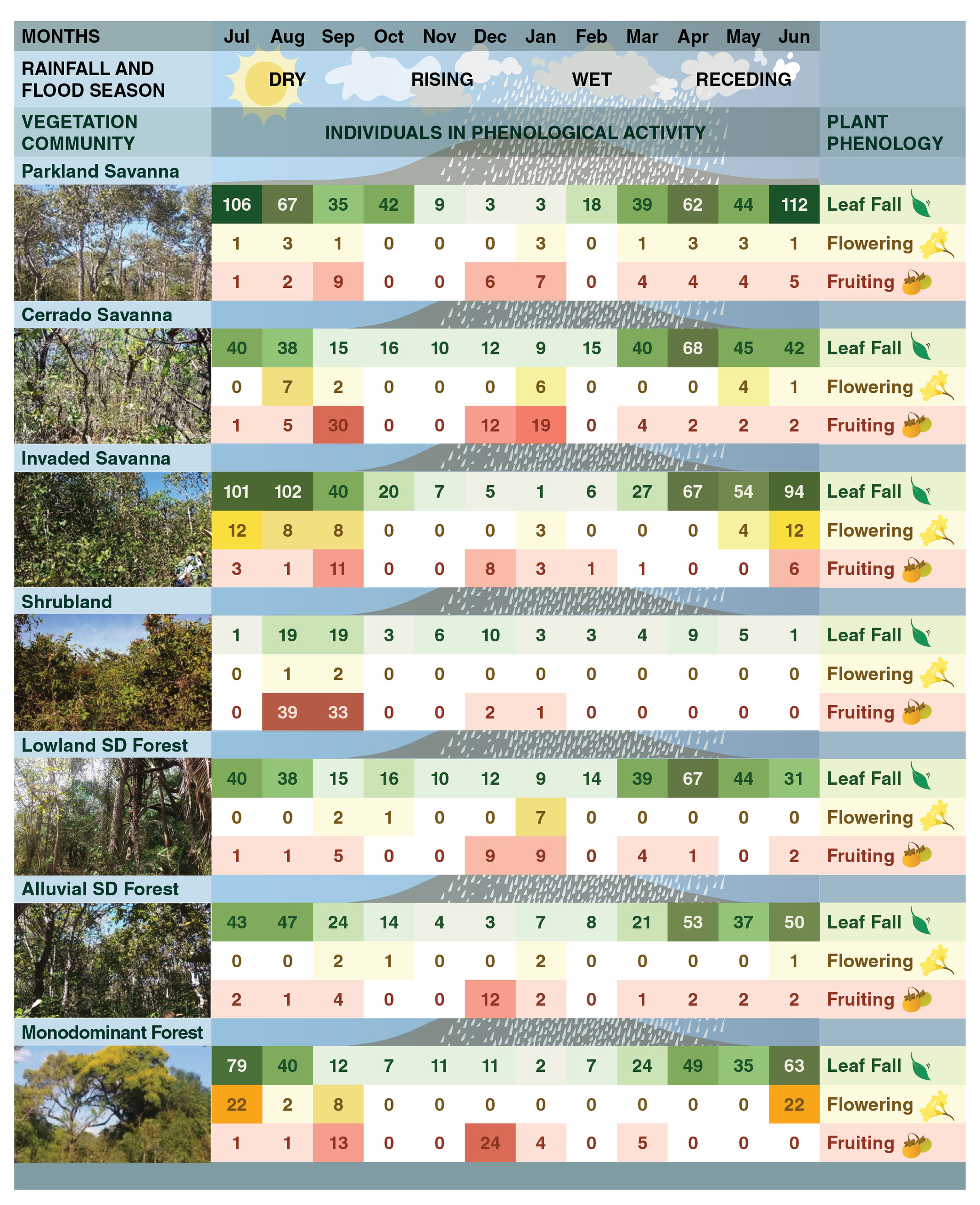

Supplement: S1 Fig — The color gradient illustrates periods of high activity (intense colors) and low activity (softer colors) among individuals. (TIF) [file pone.0316011.s001.tif]

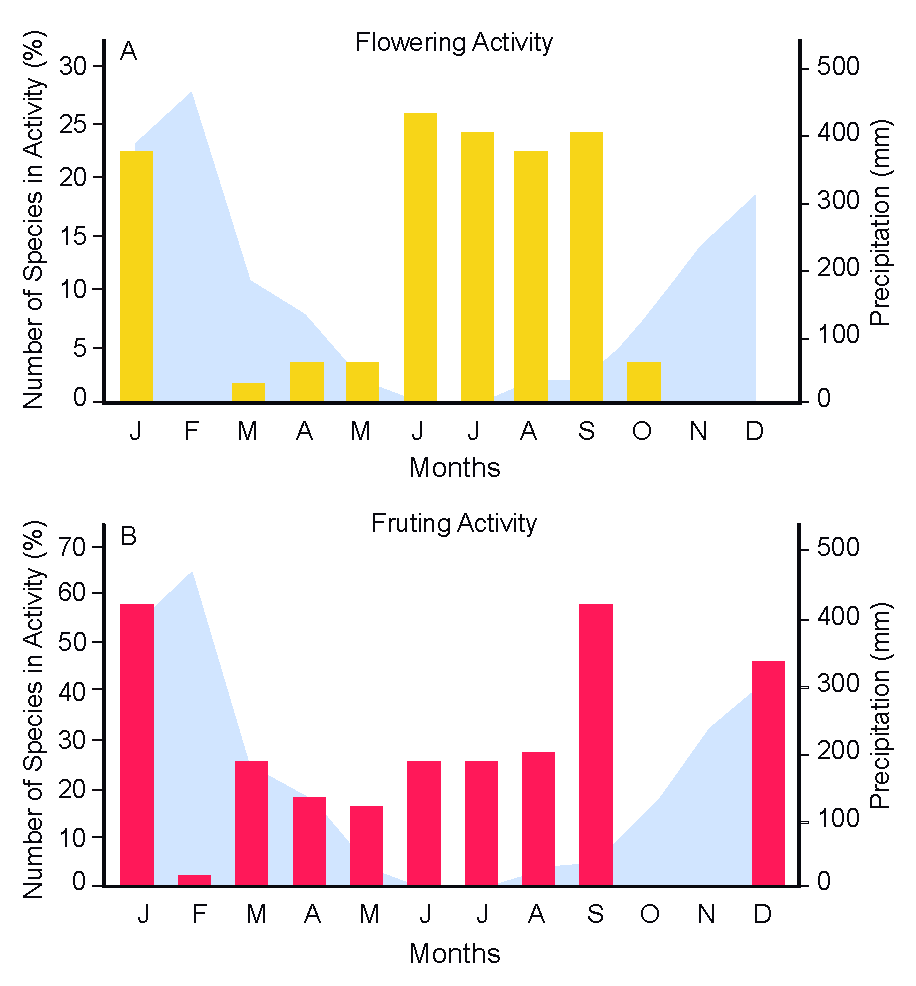

Supplement: S2 Fig — (TIF) [file pone.0316011.s002.tif]
