# Supplementary material for: Phenological cycles in the Pantanal woody communities: Responses to climate and soil moisture seasonality
Source: PLoS One. 2025 Feb 7;20(2):e0316011. doi: 10.1371/journal.pone.0316011 (PMC11805406; doi:10.1371/journal.pone.0316011)
Supplement: S1 Table — A total of 59 species from 28 families were recorded, totaling 193 individuals in 7,200 m2. The table presents the occupied communities and the months of the year (from 1 to 12, corresponding to January to December) for the activities of leaf fall, flowering, and fruiting of each species. Leaf fall activity is considered when it is above or equal to 50%. PL < 50%: Partial Leaf Fall (less than 50%); LY: Leaf Fall Lost Year-round (≥ 50%). Communities: Cerrado Savanna (CS), Invaded Savanna (IS), Parkland Savanna (PS), Shrubland (SHB), Lowland Seasonal Semideciduous Forest (LSDF), Alluvial Seasonal Semideciduous Forest (ASDF), and Semi-evergreen Monodominant Forest of Vochysia divergens (MF). (DOCX) [file pone.0316011.s003.docx]

| **Family/Species** | **N** | **Community Occupied** | **Period of Leaf Falling (≥50%)** | **Period of Flowering** | **Period of Fruiting** |
| --- | --- | --- | --- | --- | --- |
| **Anacardiaceae** |  |  |  |  |  |
| *Astronium fraxinifolium* Schott | 13 | LSDF, ASDF, CE | 08-10, 12 | 8 | 01,06-07, 09 |
| *Spondias mombin* L. | 2 | ASDF | 04-05 | - | - |
| **Apocynaceae** |  |  |  |  |  |
| *Aspidosperma cuspa*  (Kunth) S.F.Blake | 25 | LSDF, ASDF | 01-05, 08-12 | 09-10 | 12 |
| *Aspidosperma cylindrocarpon* Müll.Arg. | 2 | LSDF | 03-05, 11-12 | - | - |
| **Arecaceae** |  |  |  |  |  |
| *Attalea phalerata* Mart. ex Spreng. | 16 | LSDF, ASDF | 04-05, 08-10 | 06 | 01, 08-09 |
| *Bactris glaucescens* Drude | 2 | IS | PL <50% | - | - |
| *Copernicia alba* Morong ex Morong &  Britton | 1 | PS | 06-09 | - | - |
| **Bignoniaceae** |  |  |  |  |  |
| *Jacaranda cuspidifolia* Mart*.* | 3 | ASDF, CE | 03-10, 12 | - | - |
| *Handroanthus heptaphyllus* (Vell.)  Mattos | 51 | ASDF, CE, PS, IS | 02-11 | 01, 06-09 | 01,08-09,12 |
| *Tabebuia aurea*(Silva Manso) Benth. &  Hook. f. ex S. Moore | 53 | LSDF,ASDF,CE,PS,IS | LY | 01,08 | 01,03,08-09,12 |
| *Tabebuia roseoalba* (Ridl.) Mattos | 8 | LSDF,ASDF,CE | 03-04, 06-09 | - | 09 |
| **Boraginaceae** |  |  |  |  |  |
| *Cordia glabrata* (Mart.) A.DC. | 8 | LSDF,ASDF,CE | 02, 04-09, 11 | - | - |
| **Celastraceae** |  |  |  |  |  |
| *Salacia elliptica* (Mart. ex Schult.) G.Don | 9 | ASDF,IS | 01-05, 08-11 | 01 | 09 |
| **Chrysobalanaceae** |  |  |  |  |  |
| *Leptobalanus parvifolius* (Huber) Sothers  & Prance | 33 | LSDF,MF, PS | 02-12 | 06-07 | 01, 03, 08-09, 12 |
| **Clusiaceae** |  |  |  |  |  |
| *Garcinia brasiliensis* Mart. | 4 | LSDF, ASDF | PL <50% | - | 06-07 |
| **Combretaceae** |  |  |  |  |  |
| *Combretum lanceolatum* Pohl ex Eichler | 58 | CE,MF,SHB,IS | 01-05, 08-12 | 06-09 | 1,08-09,12 |
| *Combretum leprosum*Mart*.* | 11 | LSDF | 02, 04-05, 07-09 | 01, 07 | - |
| *Combretum rotundifolium* Rich*.* | 3 | MF, SHB | 02-05, 08-09 | - | 08-09, 12 |
| **Dilleniaceae** |  |  |  |  |  |
| *Curatella americana*L. | 10 | ASDF, LSDF,CE | 01-10, 12 | 01,08 | 01, 09 |
| **Ebenaceae** |  |  |  |  |  |
| *Diospyros lasiocalyx* (Mart.) B.Walln. | 9 | ASDF,CE,IS | 01-02, 04-08, 10, 12 | - | 01, 03 |
| **Erythroxylaceae** |  |  |  |  |  |
| *Erythroxylum anguifugum*Mart*.* | 50 | ASDF,CE,MF, PS, IS | 02-10, 12 | 01, 06-07 | 01,04-09,12 |
| *Erythroxylum suberosum* (A.St.-Hil.) | 8 | ASDF | 01-11 | - | 12 |
| **Euphorbiaceae** |  |  |  |  |  |
| *Alchornea discolor* Poepp*.* | 13 | ASDF,CE,MF,PS | 02-08, 10-12 | 09 | 12 |
| **Fabaceae** |  |  |  |  |  |
| *Anadenanthera colubrina* (Vell.) Brenan | 17 | LSDF | 01-05, 08-09, 11-12 | - | 01, 09, 12 |
| *Cassia grandis  L.f.* | 7 | IS | 04-11 | 06-09 | 09 |
| *Dipteryx alata* Vogel | 4 | LSDF,ASDF | 03-10 | 09 | 04-07 |
| *Hymenaea courbaril* L*.* | 4 | LSDF | 03-08, 11 | - | 01 |
| *Machaerium hirtum* (Vell.) Stellf. | 76 | LSDF,CE,PS,IS | 01-11 | 01, 03-09 | 01,03-07,09,12 |
| *Peltophorum dubium* (Spreng.) Taub. | 4 | LSDF | 01-05, 11-12 | - | - |
| *Tachigali aurea* Tul. | 1 | LSDF | PL <50% | 01 | 01, 09 |
| *Zygia inaequalis* (H.B.K.) Pitt. | 13 | LSDF,ASDF | 03-11 | - | 09 |
| **Lamiaceae** |  |  |  |  |  |
| *Vitex cymosa* Bertero ex Streng. | 3 | ASDF | 05-08 | - | 01,12 |
| **Lauraceae** |  |  |  |  |  |
| *Ocotea suaveolens* (Meisn.) Benth. &  Hook. f. ex Hieron. | 1 | LSDF | 06-07 | - | 01, 03, 12 |
| *Nectandra amazonum* Nees | 3 | MF | 03 | - | 12 |
| **Malpighiaceae** |  |  |  |  |  |
| *Byrsonima crassifolia* (L.) Kunth | 3 | LSDF, ASDF | PL <50% | - | - |
| *Byrsonima cydoniifolia* A.Juss. | 7 | CE,PS | 04-05, 10 | 01,04-05, 08-09 | 01, 04-06,09 |
| **Malvaceae** |  |  |  |  |  |
| *Guazuma ulmifolia* Lam. | 16 | ASDF, IS | 03, 06-10 | - | 01,06-09 |
| *Pseudobombax marginatum* (A.St.-Hil., Juss. & Cambess.) A.Robyns | 7 | ASDF,CE | 03-10 | - | 04-05, 12 |
| **Melastomataceae** |  |  |  |  |  |
| *Mouriri guianensis* Aubl. | 62 | LSDF,ASDF,CE,MF,PS,IS | 02-10, 12 | 01, 06-07,09-10 | 01-02,09,12 |
| **Meliaceae** |  |  |  |  |  |
| *Trichilia catigua* A.Juss. | 20 | LSDF,ASDF,MF, CE | 03-10 | 01 | 01-02,06-08 |
| **Moraceae** |  |  |  |  |  |
| *Brosimum gaudichaudii* Tréchul | 4 | PS,IS | 06-07, 10 | 06-07, 09 | 06-07, 09 |
| *Brosimum lactescens* (S.Moore)  C.C.Berg | 5 | LSDF,MF | 01, 06-09 | - | 04-05 |
| *Ficus insipida* Willd*.* | 1 | LSDF | 03-08 | - | - |
| **Myrtaceae** |  |  |  |  |  |
| *Eugenia florida* DC. | 33 | ASDF,CE,MF | 01-11 | 07, 09 | 01,03-05, 08-09, 12 |
| *Myrcia tomentosa* (Aubl.) DC. | 16 | ASDF, LSDF,CE,MF | 03-10, 12 | 06-07 | 01,03,12 |
| *Psidium guineense* Sw. | 7 | CE,MF,PS | 01-08, 11-12 | - | 01, 09 |
| **Nyctaginaceae** |  |  |  |  |  |
| *Neea hermaphrodita* S.Moore | 1 | LSDF | 06-09 | - | - |
| **Polygonaceae** |  |  |  |  |  |
| *Coccoloba cujabensis Wedd.* | 1 | ASDF | PL <50% |  | 01 |
| *Triplaris americana* L. | 7 | ASDF, MF | 04-07 | - | - |
| *Triplaris gardneriana* Wedd*.* | 19 | ASDF,PS, SHB, IS | LY | 06-07 | 03,09,12 |
| **Rubiaceae** |  |  |  |  |  |
| *Duroia duckei*Huber | 7 | ASDF,MF | 03-09 | 06-07 | 12 |
| *Genipa americana* L. | 12 | LSDF,ASDF,CE,IS | 03-08 | 01,06,08 | 01,09,12 |
| *Sphinctanthus microphyllus K.Schum.* | 1 | SHB | PL <50% |  |  |
| **Sapindaceae** |  |  |  |  |  |
| *Cupania vernalis* Cambess*.* | 5 | ASDF | 04, 06-10 | - | 01 |
| *Dilodendron bipinnatum* Radkl*.* | 1 | LSDF | 04-05 | - | - |
| *Magonia pubescens* A.St.-Hil. | 9 | LSDF,ASDF | 02-10, 12 | - | 01 |
| **Siparunaceae** |  |  |  |  |  |
| Siparuna brasiliensis (Spreng.) A.DC. | 2 | IS | 03-05, 07, 09-10 | - | 01 |
| **Vochysiaceae** |  |  |  |  |  |
| *Callisthene fasciculata* Mart*.* | 118 | LSDF,ASDF,CE,PS | 01, 03-10 | 01, 08-09 | 01,03-05,09,12 |
| *Vochysia divergens*Pohl | 94 | MF,SHB,PS,IS | LY | 01, 06-09 | 01, 08-09,12 |

**S1 Table. Woody species composition and vegetative and reproductive phenology of seven savanna and forest communities of the northern Pantanal. A total of 59 species from 28 families were recorded, totaling 193 individuals in 7,200 m². The table presents the occupied communities and the months of the year (from 1 to 12, corresponding to January to December) for the activities of leaf fall, flowering, and fruiting of each species. Leaf fall activity is considered when it is above or equal to 50%. PL < 50%: Partial Leaf Fall (less than 50%); LY: Leaf Fall Lost Year-round (≥ 50%). Communities: Cerrado Savanna (CS), Invaded Savanna (IS), Parkland Savanna (PS), Shrubland (SHB), Lowland Seasonal Semideciduous Forest (LSDF), Alluvial Seasonal Semideciduous Forest (ASDF), and Semi-evergreen Monodominant Forest of *Vochysia divergens* (MF).**
